# Learning from picture books: Infants’ use of naming information

**DOI:** 10.3389/fpsyg.2014.00144

**Published:** 2014-02-25

**Authors:** Melanie Khu, Susan A. Graham, Patricia A. Ganea

**Affiliations:** ^1^Department of Psychology, University of CalgaryCalgary, AB, Canada; ^2^Institute of Child Study, Ontario Institute for Studies in Education, University of TorontoToronto, ON, Canada

**Keywords:** symbolic understanding, transfer of learning, labels, representation, infants

## Abstract

The present study investigated whether naming would facilitate infants’ transfer of information from picture books to the real world. Eighteen- and 21-month-olds learned a novel label for a novel object depicted in a picture book. Infants then saw a second picture book in which an adult demonstrated how to elicit the object’s non-obvious property. Accompanying narration described the pictures using the object’s newly learnt label. Infants were subsequently tested with the real-world object depicted in the book, as well as a different-color exemplar. Infants’ performance on the test trials was compared with that of infants in a no label condition. When presented with the exact object depicted in the picture book, 21-month-olds were significantly more likely to attempt to elicit the object’s non-obvious property than were 18-month-olds. Learning the object’s label before learning about the object’s hidden property did not improve 18-month-olds’ performance. At 21-months, the number of infants in the label condition who attempted to elicit the real-world object’s non-obvious property was greater than would be predicted by chance, but the number of infants in the no label condition was not. Neither age group nor label condition predicted test performance for the different-color exemplar. The findings are discussed in relation to infants’ learning and transfer from picture books.

## INTRODUCTION

In Western societies, picture books are amongst the most common symbolic media that infants and young children encounter in their daily lives. Over the second year of life, infants in these cultures spend considerable time in shared picture book reading interactions with their parents (Payne et al., 1994; Gelman et al., 1998; Karrass et al., 2003). For example, in a recent large-scale survey, parents reported spending an average of 25 min per day reading with their 6- to 23-month-old infants (Rideout, 2011).

It is widely assumed that infants, like older children, learn about the world from these picture book interactions. Previous research has established that, by preschool age, children understand the referential nature of pictures and will use them both as symbols and sources of information about the entities they represent (e.g., DeLoache, 1991; DeLoache and Burns, 1994; Harris et al., 1997; Callaghan, 1999, 2000; Callaghan and Rankin, 2002). For example, by 4 years of age, children can learn new biological facts from picture books and transfer this information to real animals (Ganea et al., 2011).

Recent evidence indicates that symbolic understanding of pictures emerges in the second year of life (e.g., Preissler and Carey, 2004; Simcock and DeLoache, 2006; Ganea et al., 2008, 2009; Keates et al., in press) and that under supportive circumstances, infants can transfer simple information from depicted to real-world objects. For example, infants as young as 15-months of age can extend newly learnt labels from objects depicted in picture books to their real-world referents (Preissler and Carey, 2004; Ganea et al., 2008, 2009). Children aged 18-, 24-, and 30-months will also imitate an action sequence depicted in a picture book on novel real-world objects (Simcock and DeLoache, 2006, 2008; Simcock and Dooley, 2007; Simcock et al., 2011). Although infants are generally able to learn new information from picture books, their transfer of information from picture books to the real world is influenced by a number of factors, including the iconicity of the pictures (Simcock and DeLoache, 2006; Ganea et al., 2008, 2009) and the similarity between context or stimuli at encoding and test (Simcock and Dooley, 2007). A recent study by Keates et al. (in press) provided an important extension to the literature by demonstrating that 13-, 15-, and 18-month-old infants can learn about depicted objects’ hidden properties and subsequently transfer this knowledge to the real world. This ability, however, was relatively tenuous among individual infants - even at 18-months, approximately half of infants did not attempt to elicit the hidden properties. Taken together, the results of these studies raise the possibility that infants do not learn as much from parent-child picture book interactions as has generally been assumed, and that their ability to transfer this knowledge to the real world may be fairly limited. A question that emerges then is whether it is possible to improve infants’ transfer of learning from picture books by providing them with supporting information.

The goal of the present study was to examine whether providing a label for a depicted object facilitates infants’ transfer of information about that object’s properties from picture books to the real world. Using the hidden property paradigm of Keates et al. (in press), the present study investigated whether teaching 18- and 21-month-old infants labels for objects depicted in picture books, prior to teaching them about the objects’ properties, would help them generalize this information to the objects’ real-world referents. Understanding the conditions under which infants demonstrate more robust learning from picture books is important because, like other symbolic media, picture books enable infants to acquire information about the world indirectly. Accordingly, identifying ways to enhance infants’ ability to transfer knowledge from pictures books would afford them vastly greater opportunities for learning.

There is evidence that providing a name for depicted objects to infants in their third year enhances their appreciation of depictions’ symbolic status (e.g., Callaghan, 2000; Preissler and Bloom, 2007). For example, in a picture-object matching task, 2.5-year-olds succeeded in identifying depicted objects’ real-world referents *only* when their labels were known or when the depicted objects were labeled (Callaghan, 2000). Labeling has also been found to facilitate categorization, ostensibly by increasing the salience of object similarities (Waxman, 2008). Infants as young as 12-months of age will use shared object names to determine whether two objects belong to the same category, and continue to do so even when objects share minimal perceptual similarity (e.g., Booth and Waxman, 2002, 2003; Graham et al., 2004; Keates and Graham, 2008). In addition, it has been proposed that verbal cues, such as naming, may serve as a memory retrieval cue (Herbert and Hayne, 2000; Hayne and Herbert, 2004; Barr, 2010). For example, non-sense verbal labels have been shown to facilitate 24-month-olds’ deferred imitation from television (Barr and Wyss, 2008). Thus, previous research suggests that a label should provide infants with a cue to both the similarity between depicted and real-world objects, as well as the depictions’ symbolic function.

In the present study, infants were assigned to either a *label* condition or a *no label* condition. Using the picture book procedure of Ganea et al. (2008, 2009), infants in the label condition were taught a novel label (e.g., “blicket”) for a depicted novel object. Infants in the no label condition received equal exposure to the picture book, but were not provided with a label for the depicted object. Infants in both conditions were then shown a second picture book, in which a sequential series of pictures depicted an adult performing a target action to elicit the object’s non-obvious property (e.g., pushing on an object to make it light up). In the label condition, the newly learnt label was used to describe the object as the adult interacted with it. In the no label condition, the narration described the adult interacting with the object without the use of a label. At test, infants were presented with a real, 3D object identical to the one depicted in the picture book. They were subsequently presented with a different color exemplar of the object.

The primary question of interest was whether infants in the label group would be more likely than infants in the no label group to learn and transfer a non-obvious property from a picture book, as demonstrated by their performance of the target action on the real-world object. Further, we aimed to determine whether infants in the label condition would be more likely than infants in the no label condition to generalize their learning to the different color exemplar. The ability to generalize knowledge about an object’s non-obvious property to a novel exemplar would indicate more robust learning, given that infants would have to overcome even greater perceptual differences between the depicted object and its real-world referent. An additional question we sought to address was whether there would be age-related differences in the effectiveness of naming information. Accordingly, both 18- and 21-month-olds were tested. Age-related changes in infants’ ability to benefit from naming information were anticipated based on documented age-related constraints on infants’ memory flexibility (Barr, 2013) and working memory (Garon et al., 2008), as well as previous research demonstrating changes in infants’ symbolic use of pictures between 18- and 24-months of age (e.g., Simcock and DeLoache, 2006; Ganea et al., 2009).

## MATERIALS AND METHODS

### PARTICIPANTS

Participants were 96-, 18-, and 21-month-old infants. Infants in each age group were assigned to one of two conditions: the *label* condition or the *no label* condition. Infant demographic information is presented in **Table 1**. An additional 29 infants were tested, but were excluded from the final sample due to excessive fussiness (*n* = 21), parental interference (*n* = 1), or failure to learn at least one label (*n* = 5) in the label condition. Participants were recruited at local trade shows and through community advertisement. All infants were born full-term and came from homes in which English was the primary language spoken. This study was approved by the Conjoint Ethics Research Board at the University of Calgary. Parental consent for participation was obtained in writing prior to the testing session.

**Table 1:** Infant demographic information as a function of age and condition.

	Age		CDI		Books	Gender
	M (SD)	Range	M (SD)	Range	M (SD)	
**18-month-olds**
No label condition	18.6 (0.2)	18.1–18.9	142 (131)	9–438	5.5 (4.5)	12 Female 12 Male
Label condition	18.5 (0.2)	18.1–18.9	67 (53)	8–199	5.0 (4.0)	10 Female 12 Male
**21-month-olds**
No label condition	21.6 (0.2)	21.1–22.0	150 (108)	12–393	4.5 (3.7)	11 Female 14 Male
Label condition	21.5 (0.3)	21.0–22.0	212 (122)	30–428	5.0 (4.7)	12 Female 13 Male

*
Age = age in months; CDI, number of words produced based on parental report on the MacArthur-Bates CDI; Books, number of books parents report reading with their infant daily.
*

### MATERIALS

#### Object sets

**FIGURE 1:**
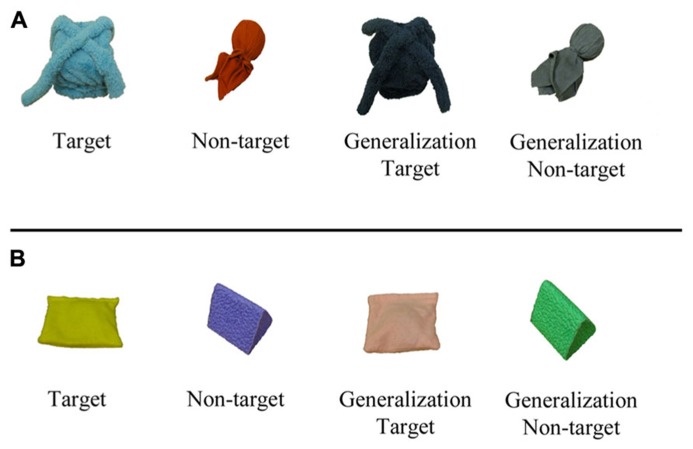
**(A)** The box object set. **(B)** The light object set.

Two object sets were used throughout the study: a light object set and a box object set (see **Figure 1**). Each set consisted of four objects: a target object, a non-target object, a generalization target exemplar, and a generalization non-target exemplar. The target box object was a square-shaped box (13 cm in width × 13 cm in length × 13 cm in height) covered with fuzzy, blue polar fleece and topped with two long pieces of the same material, crossed over one another. The box was filled with colorful ribbon, which was attached to a spring glued to the bottom of the box. When the lid of the box was lifted, the ribbon inside the box “popped up.” The generalization target exemplar was constructed identically to the target object, but was covered with black fuzzy polar fleece. The non-target object was a rubber ball (3.34 cm in diameter) covered with orange corduroy and shaped with string and sponge. The generalization non-target exemplar was identical to the non-target object, but it was covered with grey corduroy. The target light object was a push light (21 cm in width × 21 cm in length × 2.5 cm in height) covered with yellow felt. The generalization target exemplar was a push light covered with pink felt. The light inside the felt lit up when pressure was applied to the top of the object. The non-target object was a triangular prism (10 cm in width × 12 cm in length × 9 cm in height) covered with purple foam. The generalization non-target exemplar was identical to the non-target object, but it was covered with green foam.

#### Labeling phase

**FIGURE 2:**
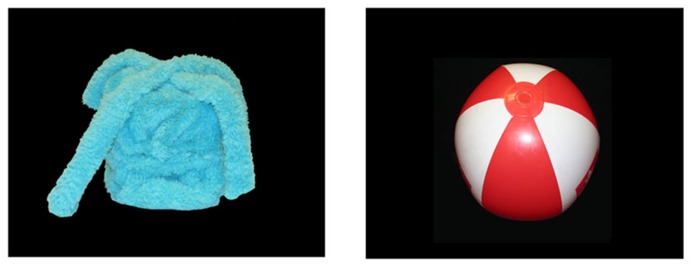
**Two pictures used in the word learning phase and the label comprehension phase.** These pictures show a ball and the box target object.

Stimuli consisted of two picture books (25 cm × 30 cm), one for each object set. Each picture book contained 14 color photographs (19 cm × 13 cm): four photos of a novel target object, four photos of a novel non-target object, and six photos of familiar objects. The same six familiar objects were used for both picture books (shoe, ball, cup, apple, bottle, car), and had labels produced by at least 90% of 18-month-old infants, as indicated by the MacArthur-Bates Lexical Developmental Norms (Dale and Fenson, 1996). Typed narration was provided below each picture. When the book was open, infants saw two pictures side-by-side (see **Figure 2**). Throughout the book, pictures of familiar and novel objects were presented on opposite pages, with the exception of the final two pages, where the novel target and non-target were presented together.

#### Label comprehension phase

Stimuli consisted of a subset of the photographs used during the labeling phase (bottle, car, ball, cup, light object target, light object non-target, box object target, box object non-target). Each photo was presented on an individual, laminated page (22 cm × 29 cm).

#### Non-obvious property phase

**FIGURE 3:**
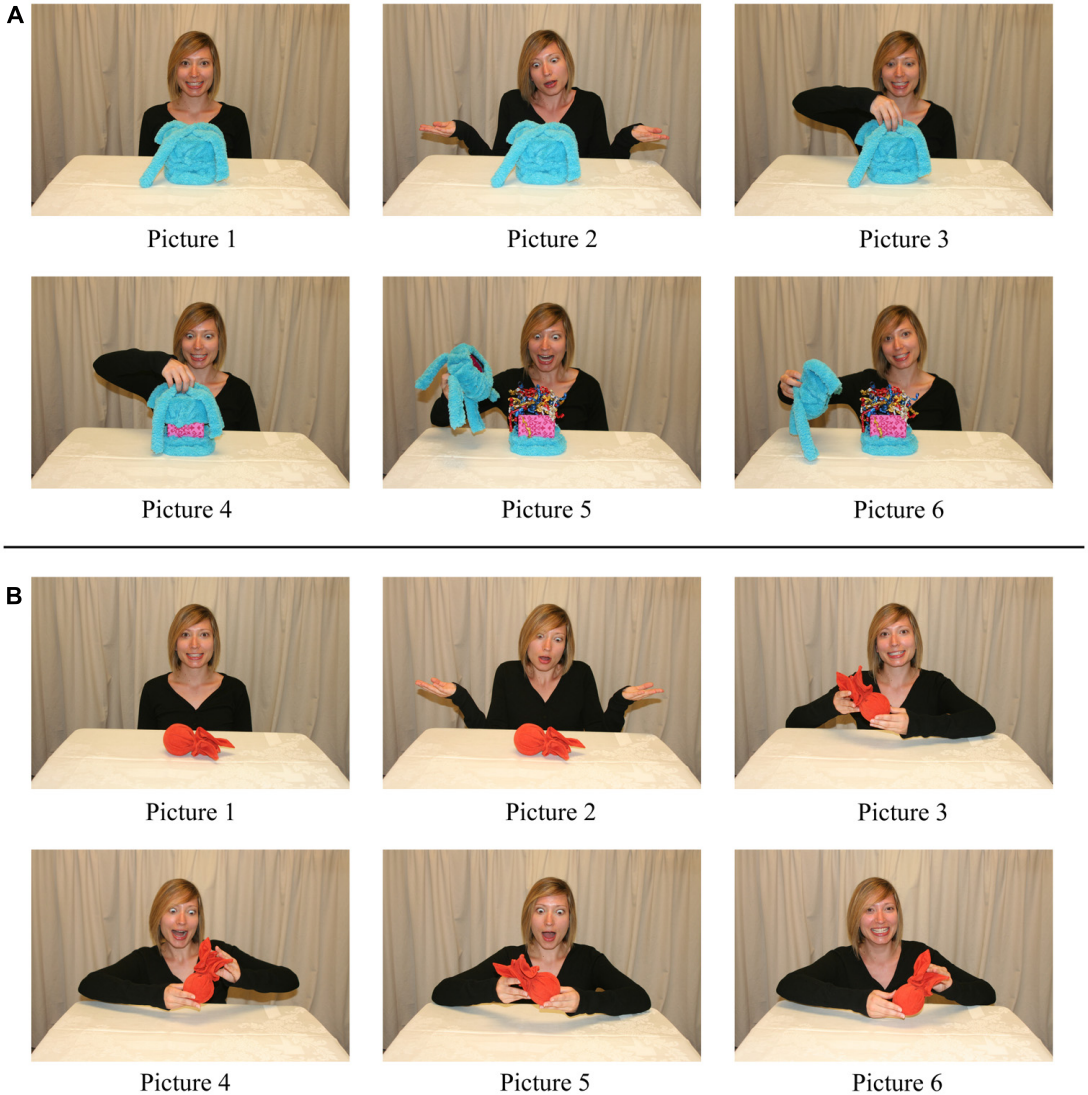
**Sequence of pictures used in the non-obvious property phase.** **(A)** The target object of the box object set. **(B)** The non-target object of the box object set. Adapted from Keates et al. (in press).

Stimuli consisted of two picture books with dimensions identical to those of the books used during the labeling phase. Each picture book contained 12 color photographs of an adult seated at a table with a novel object. In six photos, the adult was depicted with the target object and in six photos the adult was depicted with the non-target object. For the target, the adult performed an action that elicited the object’s non-obvious property, and for the non-target, the adult explored the object without performing an action on it (see **Figures 3A,B**). Each photo was presented individually, such that when the book was open, the picture was on the right side of the book. Typed narration was provided below each picture.

#### Test phase

Stimuli consisted of eight objects, four from each of the two object sets described above (i.e., the box set and the light set). The target and non-target objects were used for the extension trials and the generalization target and non-target exemplars were used for the generalization trials. A handheld stopwatch was used to time the trials.

### PROCEDURE

The infant was seated across a table from the experimenter, either in a booster chair or on the parent’s lap. The parent was instructed not to direct, prompt, or cue the infant during the task. The parent was further instructed to place objects back within reach of the infant if the infant handed the objects to them or dropped the objects on the floor. Testing consisted of two blocks of four phases: labeling phase, label comprehension phase, non-obvious property phase, and test phase. Each block corresponded to one object set (i.e., box set or light set). The order of blocks was counterbalanced across participants. For coding purposes, all sessions were recorded using a 6.1 MP Sony Digital HD video camera.

#### Labeling phase

The experimenter sat next to the child at a table, and read the typed narration while pointing to the depicted objects. For each familiar picture, the experimenter labeled the object once (e.g., “Look, it’s a car”). For the novel target object, the experimenter labeled the object three times (e.g., “Look, this is a *blicket*. Wow, it’s a *blicket*. See a *blicket*?”). For the non-target object, and the target object in the no label condition, the experimenter drew the infant’s attention to the object three times without labeling it (e.g., “Look, look at that. Wow, it’s that. See that?”). For each pair of pictures (i.e., a familiar object and novel object), the familiar object was presented first, on the left side of the book, and the novel object was presented second, on the right side of the book*. *The order in which the novel target and non-target objects were presented in the picture book was counterbalanced across infants.

#### Label comprehension phase

During this phase, the experimenter sat across the table from the infant. For infants in the label condition, the experimenter presented two pictures of familiar objects and asked the infant to indicate one of them (“Show me the car [ball, shoe, cup]”). The object requested, as well as the side on which the target picture presented, was counterbalanced across participants. If the infant did not respond, the experimenter used alternate phrases (e.g., “Where’s the car?” or “Point to the car”), until a response was elicited. If the infant did not respond to the experimenter, the experimenter instructed the parent to repeat the phrases, until a response was elicited. On subsequent trials, the experimenter asked the child to indicate the objects using whichever phrase had elicited a response. Then, to assess whether infants had learnt the novel label for the depicted target object, the experimenter presented two photographs: one of the novel target and one of the novel non-target. She then asked the infant to indicate the target (“Show me the blicket”). Infants were given positive reinforcement (e.g., “That’s right! Good job!”) when they chose the target picture and were given corrective feedback (e.g., “Remember, this one is the blicket”) when they chose the non-target. The criterion was two correct successive responses on two trials, with a maximum of four possible trials, following that used in previous research (e.g., Ganea et al., 2009).

Infants in the no label condition were also shown the pair of familiar objects and the pair of novel objects (i.e., target and non-target). Rather than being asked to indicate a specific object, infants were asked to show either one of the objects to the experimenter (“Show me one”). The experimenter prompted the infant (as described above), until the infant chose one of the objects. Regardless of the infant’s choice, the experimenter provided a neutral response (“Thank you”).

#### Non-obvious property phase

During this phase, the experimenter read the non-obvious property book to the infant in the manner described above. The infant saw a sequence of six photographs of the adult interacting with the first novel object (e.g., the target), followed by a sequence of six photographs of the adult interacting with the second novel object (e.g., the non-target). The narration for the target object described the adult eliciting the object’s non-obvious property by performing the target action.**In the label condition, the pictures were described using the object’s newly learnt label (i.e., the label that was taught during the labeling phase). In the no label condition, the pictures were described without the use of a label to refer to the target object. In both conditions, the narration for the non-target object described the adult exploring the object without performing an action. The narration was approximately equivalent in length for the target and non-target picture sequences in order to equate the attention paid to both depicted objects. The order of the six pictures within each sequence was fixed, but the order of presentation of the sequences (i.e., target vs. non-target sequence presented first) was counterbalanced across infants.

#### Test phase

During this phase, the experimenter sat across the table from the infant. For the extension trial, she simultaneously placed the exact target and non-target objects that were depicted in the book on the table, out of reach of the infant. In the label condition, she introduced the objects to infants using the newly learnt label (e.g., “Look. There’s a blicket here. Now you get to play!”). In the no label condition, she introduced the objects by substituting the word “toy” for the object label (e.g., “Look. There’s a toy here. Now you get to play!”). She then moved the objects within the infant’s reach and gave the infant the opportunity to explore the objects for 20 s.

After 20 s had elapsed, the experimenter retrieved the two objects and intitiated the generalization test trial. The experimenter simultaneously placed the generalization target and non-target exemplars on the table, out of reach of the infant. She introduced the objects using the same newly learnt label (e.g., “Look. There’s a blicket here. Your turn again!”) for infants in the label condition, or substitued the word “toy” for infants in the no label condition. She then placed the objects within the infant’s reach. The infant were again given 20 s to explore the two objects. If, over the course of the 20 s exploration period, the infant could no longer reach the object, the experimenter or parent re-placed the object in front of the infant within his or her reach.

The extension test trial was always presented before the generalization test trial. Consistent with previous research examing children’s transfer from picture books (e.g., Ganea et al., 2008), it was reasoned that presenting the test trials in this order would help to clarify interpretation of infants’ performance. That is, our primary objective was to investigate infants’ transfer from picture books, and the clearest test of this tranfer was the extension trial. If the generalization test were presented first, and infants failed to demonstrate evidence of transferring the depicted property, it would be unclear whether they were (a) unable to generalize to a novel exemplar, or (b) unable to tranfer from the picture book to a real object more generally. As a result, it was determined that having the extension trial precede the generalization would simplify the interpretation of infants’ performance, despite limiting conclusions that could be drawn about infants’ generalization (i.e., the extent to which infants can generalize non-obvious properties to novel exemplars, in the absence of experiencing a more similar exemplar first).

Once the first block of trials was completed, the second block of trials was administered for the other object set. Following the testing session, the parent was asked to complete the MacArthur-Bates Communicative Development Inventory: Words and Sentences (CDI; Fenson et al., 2007), a measure of productive vocabulary. The parent was also asked to indicate the number of picture books the infant and parent read together per day.

### CODING AND RELIABILITY

Infants’ attempts to elicit the target objects’ non-obvious properties were coded offline by trained coders, unaware of the experimental hypotheses and participants’ condition. The target action for the box object set was defined as forcefully pulling upward on the material on top of the object. Picking at or touching the material on the top of the object without lifting or pulling the material, lifting the long pieces of material on the top of the object without using force (e.g., lightly holding them a vertical position), or shaking or squeezing the object, were not coded as target actions. The target action for the light object set were defined as hitting, pushing on, or tapping the object with the hand or fingers using a swift “tap-like” motion. Actions performed on the excess felt around the push light, rather than on the top or side of the felt-covered push light itself, were not coded as target actions. Lightly resting a hand on top of the object, without pushing or applying pressure, or touching the object in order to feel or poke it, were also not coded as target actions. For both object sets, actions performed in order to pick up, throw, move the object closer to oneself, or pass the object to either the parent or the experimenter, were not coded as target actions.

Coders also recorded the amount of time infants spent examining the target or non-target objects. Examination time was used as a measure of infants’ interest in the objects, and was defined as the number of seconds spent looking at, or looking at and touching, the objects.

An additional coder, unaware of the experimental hypotheses and condition, coded 20% of the videos. Inter-rater reliability for target actions on target objects was high (κ = 0.968). Inter-rater reliability for examination time coding was also high (intraclass correlation coefficient = 0.980).

### PREDICTIONS

First, we predicted that infants in the label condition would be more likely than infants in the no label condition to perform the target actions on the real-world objects for both the extension and generalization trials. Furthermore, we expected that the facilitative effects of the label might be more pronounced for the generalization trial, because of the challenge inherent in transferring to a more perceptually dissimilar exemplar. Second, we predicted that there would be age-related changes in infants’ ability to benefit from naming information, with greater differences between the label and no label conditions at 21-months than at 18-months. Finally, it was anticipated that infants in the label condition, across both age groups and test trials, would spend more time examining the target object relative to the non-target object, but that infants in the no label condition would examine the target and non-target objects equally.

## RESULTS

### PRELIMINARY ANALYSES

First, we assessed comprehension of the object labels for infants in the label condition to ensure that infants were in fact mapping the novel label to the depicted target objects so that any observed differences in the performance of the label and no label condition could be attributed to differences in access to naming information. Infants who had not learnt at least one of the object labels were excluded from subsequent analyses (*n* = 5). Overall, 24 of the 47 infants assigned to the label condition demonstrated evidence of learning the novel labels for both targets (i.e., depicted light and box target objects) and 23 of the infants learnt the label for one of the two targets.

Next, within each age group, we examined infants’ productive vocabulary and exposure to picture books in order to determine whether these differed between conditions. All analyses were performed using IBM SPSS Statistics software (version 20; IBM Corp., Chicago, IL, USA). The number of books parents reported reading to their infant daily did not vary by age or condition *p*’s**> 0.707. The 21-month-old infants had larger productive vocabularies than the 18-month-old infants, *t*(94) = 3.24, *p* = 0.002. The 18-month-olds in the no label group had higher productive vocabulary scores than the 18-month-olds in the label group *t*(30.97) = 2.56, *p* = 0.016. There was no difference in the number of words produced by infants in the label and no label condition at 21-months (*p* = 0.064).

### PRIMARY ANALYSES

Infants’ learning and transfer of non-obvious properties was analyzed in two ways. First, infants’ performance of the depicted target action on the real target object was analyzed to determine whether they had successfully transferred their learning from the depicted target to its real-world referent. Second, the time that infants spend examining the target objects relative to the non-target objects was analyzed as a measure of infants’ interest in the target objects during the test trials.

#### Target actions

Sequential logistic regression analyses were conducted to assess the prediction of test outcome (i.e., whether or not infants performed target actions to elicit objects’ non-obvious properties). Only one 18-month-old performed two target actions on the extension trial (across the two testing blocks), and only four 18-month-olds (two per label condition) performed two target actions on the generalization trial (across the two testing blocks). As a result, the number of cases per cell was not sufficient to support a multinomial logistic regression approach. Test outcome was accordingly classified dichotomously. That is, infants were given credit for performing a target action on either the light or the box object target object for the extension trial, and were given credit for performing a target action on either the light or the box generalization target exemplar for the generalization trial. If infants performed target actions on both sets, no additional credit was given. **Table 2** displays the test outcome by condition and age group contingency table for the extension trial. **Table 3** displays the test outcome by condition and age group contingency table for the generalization trial. There was no significant difference between infants’ performance of target actions on the light target and infants’ performance of target actions on the box object (McNemar test, *p* = 0.132).

**Table 2:** Extension trial: Learning as a function of condition and age group.

		Attempt to elicit property		
Age group	Condition	No	Yes	Total
**18-month-olds**
	No label	14	10	24
	Label	11	11	22
	Total	25	21	
**21-month-olds**
	No label	10	15	25
	Label	5	20	25
	Total	15	35	

**Table 3:** Generalization trial: Learning as a function of condition and age group.

		Attempt to elicit property		
Age group	Condition	No	Yes	Total
**18-month-olds**
	No label	12	12	24
	Label	10	12	22
	Total	24	24	
**18-month-olds**
	No label	11	14	25
	Label	5	20	25
	Total	16	34	

Preliminary analyses indicated that the following variables did not meaningfully contribute to the prediction of test outcome: gender, the order in which object sets were presented (i.e., light object set first vs. box object set first), the number of picture books parents reported reading with their infant daily, and the age by condition interaction term. Accordingly, these variables were excluded from subsequent analyses.

***Extension trial.*** To explore the contribution of naming to infants’ performance on the extension test trial, a sequential dichotomous logistic regression was conducted, with attempt to elicit a target object’s non-obvious property for at least one target object set (performance of a target action vs. no performance of a target action) as the dependent variable (**Table 4**). Age group (18-month-olds vs. 21-month-olds) was entered on step 1. Condition (label condition vs. no label condition), was entered on step 2. Productive vocabulary (as indicated by parental report on the MCDI) was entered on step 3. Inclusion of this variable helped address the between-group differences in vocabulary noted above (i.e., the difference between the productive vocabularies of 18-month-olds in the label vs. no label condition), by distinguishing and accounting for the variance explained by condition, and the variance explained by vocabulary.

**Table 4:** Logistic regression analysis predicting test performance from age group, condition, and productive vocabulary (extension trial).

Predictor	χ^2^ to remove	df	Model χ^2^
Step 1			5.90*
Age group	5.90*	1	
Step 2			8.09*
Condition	2.19	1	
Step 3			8.29*
Productive vocabulary	0.19	1	

*p < 0.05.

For step 1, the Likelihood ratio test for the overall model was significant, *χ*^2^ (1, *N* = 96) = 5.90, *p *= 0.015, indicating that compared to a constant-only model, infants’ age contributed significantly to the prediction of infants’ performance of target actions. The addition of condition to the model in step 2 did not significantly improve the model fit, *p *= 0.139. The Likelihood Ratio test for the overall model remained significant, *χ*^2^ (2, *N* = 96) = 8.09, *p *= 0.018. When productive vocabulary was added to the model in step 3, the improvement in the model fit was again not statistically significant *p *= 0.660, and the Likelihood ratio test for the overall model remained significant *χ*^2^ (3, *N* = 96) = 8.29, *p *= 0.040. The effect size of the model with all three predictors compared to the constant-only model was small, Nagelkerke = 0.111, indicating that these variables accounted for only 11.1% of the between-group variance.

**Table 5** shows regression coefficients, Wald statistics, odds ratios, and 95% confidence intervals for the odds ratios for each individual predictor. The only predictor that contributed to the prediction of whether an infant would attempt to elicit a non-obvious property by performing a target action was age, *B* = 0.97, *SE* = 0.45, *Wald*(1) = 4.64, *p* = 0.031. For infants in the 21-month-old group, the odds in favor of performing a target action on a target object were 2.65 times larger than for infants in the 18-month-old group; 70% (35/50) of infants in the 21-month-old group performed a target action compared to 46% (21/46) of infants in the 18-month-old group.

**Table 5:** Predictors of test performance on the extension trial.

Variable	OR	95% CI	*p*
Age group	2.65	[1.09, 6.42]	0.03
Condition	1.91	[0.84, 4.49]	0.14
Productive vocabulary	1.00	[1.00, 1.00]	0.66
(Constant)	0.56		0.18

*
OR, odds ratio; CI, confidence interval.*

Approximately half of infants in the no label condition performed target actions (51%, 25/49). Similarly, approximately half of infants in the Keates et al. (in press) study performed target actions (51%, 31/61). Across different age groups (i.e., 13-, 15-, 18-, and 21-months), it appears that the chance success rate (in the absence of supporting information, such as shared labels) is roughly 50%. In the present study, the number of 18-month-olds who performed target actions did not differ reliably from chance (i.e., 50%), *χ*^2^ (1, *N* = 46) = 0.35, *p *= 0.555. Conversely, the number of 21-month-olds who performed target actions was reliably higher than would be predicted by chance, *χ*^2^ (1, *N* = 50) = 8.00, *p *= 0.005. Within the 21-month-old group, more infants in the label condition performed target actions than would be predicted by chance, *χ*^2^ (1, *N* = 25) = 9.00, *p *= 0.003, however, the performance of infants in the no label condition did not differ reliably from chance, *χ*^2^ (1, *N* = 25) = 1.00, *p *= 0.317.

***Generalization trial.*** To explore the contribution of naming to infants’ performance on the generalization test trial, a second sequential dichotomous logistic regression was performed (see **Table 6**). The dependent variable and predictors, as well as the steps of the analysis, were identical to those described for the extension test trial.

**Table 6:** bf Logistic regression analysis predicting test performance from productive vocabulary, age group, and condition (generalization trial).

Predictor	χ^2^ to remove	df	Model χ^2^
Step 1			2.52
Age group	2.52	1	
Step 2			4.75
Condition	2.23	1	
Step 3			5.97
Productive vocabulary	1.22	1	

For step 1, Likelihood ratio test for the overall model was not significant, *p *= 0.113, indicating that age group did not contribute to the prediction of performance of target actions. The addition of condition in step 2 did not significantly improve the fit of the model, *p *= 0.135 and the Likelihood Ratio test remained non-significant, *p *= 0.093. The addition of productive vocabulary to the model in step 3 also did not significantly improve the fit of the model, *p *= 0.270. A test of the model with all three predictors against a constant-only model remained non-significant, *p *= 0.113, indicating that the variables, as a set, did not reliably distinguish between infants who had and had not performed target actions.

Examination of the Wald statistic for each of the individual predictors (i.e., age group condition, productive vocabulary) confirmed that none of these variables significantly contributed to the prediction of infants’ performance, *p*s > 0.127. Thus, unlike the extension test trial, in which age group was a significant predictor of infants’ performance, for the generalization test trial none of the predictors reliably distinguished between infants who learnt and did not learn from the picture book. As in the extension trial, the number of 18-month-olds who performed target actions on the generalization trial did not differ reliably from chance (i.e., 50%), *χ*^2^ (1, *N* = 46) = 0.087, *p *= 0.768, whereas the number of 21-month-olds who performed target actions was reliably higher than would be predicted by chance, *χ*^2^ (1, *N* = 50) = 6.48, *p *= 0.011. Further examination of the 21-month-old group’s performance again revealed that more infants in the label condition performed target actions than would be predicted by chance, *χ*^2^ (1, *N* = 25) = 9.00, *p *= 0.003, but that the number of infants in the no label condition performing target actions did not differ reliably from chance, *χ*^2^ (1, *N* = 25) = 0.36, *p *= 0.549.

#### Examination time

In an additional set of analyses, the time that infants spent examining the target objects over the course of the test trials was analyzed. Examination time for target objects was proportionalized by dividing the number of seconds infants spent interacting with the target object by their total examination time for both the target object and non-target object. The proportion of examination time for each object set (i.e., the light object set and box object set) was averaged to yield one mean target object examination time score for each trial type (i.e., extension and generalization). Mean proportion examination times for the target objects, separated by trial, condition, and age group are presented in **Table 7**.

**Table 7:** Mean proportion examination times for the target object by condition and age group (extension and generalization trials).

		Test trial	
		Extension	Generalization
Age group	Condition	*M* (SD)	*M* (SD)
**18 Months**

	No label	0.45 (0.24)	0.56 (0.22)
	Label	0.45 (0.16)	0.55 (0.18)
	Mean^a^	0.45 (0.20)	0.56 (0.20)
**21 Months**
	No label	0.56 (0.20)	0.60 (0.17)
	Label	0.57 (0.16)	0.62 (0.19)
	Mean^a^	0.57 (0.18)	0.61 (0.18)

a Averaged across condition.

To examine whether infants’ examination times for the target objects varied as a function of condition, age group, and test trial, a 2 (*Condition*: Label vs. No Label) × 2 (*Age Group*: 18-month-olds vs. 21-month-olds) × 2 (*Test Trial*: Extension vs. Generalization) mixed factor ANOVA was conducted with test trial as a repeated measure. This analysis revealed a significant main effect of age group, *F*(1,92) = 6.78, $\eta_{p}^{2}$ = 0.07, *p *= 0.011, with 21-month-old infants spending significantly more time examining the target objects on the test trials than 18-month-old infants. There was also a significant main effect of test trial, *F*(1,92) = 10.78, $\eta_{p}^{2}$ = 0.11, *p *= 0.001, with infants spending significantly more time examining the target objects on the generalization test trials than on the extension test trials. There was no effect of condition and there were no two-way or three-way interactions involving age group, test trial, or condition, *p’*s**> 0.074. These results suggest that infants in the label and the no label conditions were equally interested in the target objects. As a group, the 21-month-olds were significantly more interested in the target objects than the 18-month-olds, and across age groups, infants were more interested in the generalization target exemplars than the exact target objects depicted in the picture books.

## DISCUSSION

The present study investigated whether naming would facilitate infants’ transfer of complex information from picture books to the real world, as well as potential age-related differences in the effectiveness of this verbal cue. When infants were presented with the exact object depicted in the picture book (the extension trial), age was an important predictor of performance of target actions. Specifically, for infants in the 21-month age group, the odds of attempting to elicit a target object’s non-obvious property were almost 2.65 times greater than for infants in the 18-month age group. For the extension trial, the presence of label information did not influence 18-month-olds’ performance; the number of 18-month-olds who performed target actions in both the label and no label condition did not differ reliably from chance. Similarly, the number of 21-month-olds who performed target actions in the no label condition did not differ from chance. Thus, the only condition in which the number of infants performing target actions was greater than would be predicted by chance was the 21-month-old label condition. When presented with a different color exemplar of the object depicted in the picture book (generalization trial), neither age group nor label condition distinguished between the infants who performed target actions and those who did not perform target actions.

### EXTENSION TRIAL

On the extension trial, older infants were more likely than younger infants to transfer objects’ non-obvious properties from picture books to real-world objects, a finding consistent with previous research demonstrating increases in infants’ symbolic understanding of pictures over the second year of life (e.g., Simcock and DeLoache, 2006; Simcock and Dooley, 2007; Ganea et al., 2009). These age-related differences have been attributed to both children’s emerging symbolic capacity, as well as greater flexibility in mental representations (e.g., Simcock and DeLoache, 2006; Barr, 2013). Interestingly, the age-related changes in infants’ performance in the current research differ from the findings of Keates et al. (in press), where infants 13-, 15-, and 18-months of age did not differ significantly in their attempts to elicit the depicted non-obvious property with the real target object. One possibility is that between 13- and 18-months of age, infants’ symbolic understanding of picture books is fairly comparable, with this understanding then developing rapidly between 18- and 21-months of age. Another possibility is that the age effects of the present study can be partially attributed to the facilitation observed in the 21-month-old label condition. That is, as a result of the greater number of infants in the 21-month-old label condition performing target actions, the overall number of 21-month-olds performing target actions was significantly greater than the number of 18-month-olds.

The finding that labels facilitated 21-month-olds’ transfer from picture books on the extension trial is consistent with other research that has shown that verbal cues improve imitation from not only picture books, but also television, another 2D symbolic medium (e.g., Barr and Wyss, 2008; Barr, 2010; Seehagen and Herbert, 2010; Simcock et al., 2011). In contrast to the facilitation observed at 21-months, the presence or absence of naming information did not appear to influence infants’ performance on the extension trial at 18-months. This was unexpected, given that previous research has documented the facilitative effects of naming in other types of tasks, as well as with even younger infants (e.g., Booth and Waxman, 2002, 2003; Graham et al., 2004; Keates and Graham, 2008; Waxman, 2008; Herbert, 2011). The lack of facilitation reported here likely resulted from two factors. One is the cognitive demands placed on infants in the label group: they had to encode and form a representation of the target object and its label, and then hold this information in mind while learning how to elicit the object’s non-obvious property. In order to succeed on the test trials, infants then had to simultaneously activate the representation of the object, its label, its non-obvious property, and how to elicit this property. Finally, infants had to select the correct target object and perform the appropriate target action. It is possible that the task demands taxed 18-month-olds’ cognitive resources, interfering with their ability to use the naming information that was provided^1^. The second factor is the well-documented challenges experienced by infants faced with the task of transferring complex information from 2D to 3D contexts (Barr, 2010, 2013). Studies examining infants’ imitation of action sequences from pictures have consistently found that 18-month-olds who are presented with a depicted, three-step action sequence do not re-enact the entire sequence (Simcock and DeLoache, 2006; Simcock and Dooley, 2007), and further, have difficulty producing the target actions in the correct order (Simcock et al., 2011).

What, beyond the general effect of age, might account for the observed changes in the effectiveness of the naming information between 18- and 21-months? First, 21-month-olds possess more advanced representational systems than 18-month-olds, including language and memory systems, as well as more developed perceptual and motor systems (Barr, 2010). It should be noted however, that infants’ productive vocabulary did not uniquely predict performance, suggesting that infants’ language proficiency was only one of a number of factors contributing to their performance. Second, 21-month-olds have had more exposure and interaction to symbols in their daily lives, and thus they may have had more opportunities to clarify the symbolic relations between symbols and their referents. Accordingly, they may have a more robust understanding of the symbolic nature of pictures. Finally, 21-month-olds’ overall cognitive processing is likely faster, and more flexible than that of younger infants, allowing them to integrate perceptual and linguistic input more quickly (Garon et al., 2008; Barr, 2010).

Similar age-related changes in the ability to benefit from naming information have been reported in studies examining the transfer from touchscreens or television sources to real-world objects. Specifically, a recent study by Zack et al. (2013), examining 15-month-old infants’ imitation from touch screens, failed to find facilitation from shared labels. As in the present study, their task was relatively complex, required infants to transfer information from a 2D symbolic medium to a 3D real-world object, and found that the addition of object labels had no effect on infants’ transfer. At 24-months, however, non-sense verbal labels provided by either parents or voice-overs were shown to enhance infants’ imitation from television (Barr and Wyss, 2008). The parallels in age-related differences across different kinds of 2D to 3D transfer support the notion that developments in general cognitive abilities such as working memory and memory flexibility, as well as developments in representational and symbolic systems, influence the effectiveness of verbal cues such as naming information.

### GENERALIZATION TRIAL

Given that the generalization exemplars were less perceptually similar to the depicted objects than the extension exemplars, it was expected that this test trial would pose a greater challenge, resulting in a greater potential to observe the facilitative effects of naming information. However, neither age group, nor label condition, nor productive vocabulary, meaningfully contributed to the prediction of infants’ performance. Contrary to the above-mentioned hypothesis, it appears as though infants were actually *more* interested in the target for this trial relative to the extension trial. As a result of the increased interest, it is possible that the relatively small effect of age became even less pronounced.

The fact that infants’ performance was similar across both the extension and generalization trial suggests that, contrary to our predictions, the generalization trial did not pose a greater challenge. It is possible that always having the generalization follow the extension removed any effects by allowing infants to extend their knowledge from the picture book to the extension target object, and from the extension target object to the generalization target object. Furthermore, it is possible that some of the 18-month-olds used their experience with the extension trial to succeed on the generalization trial, obscuring the age effects found on the extension trial. Future research could investigate whether presenting the generalization trial without the extension trial would increase the difficulty of the trial, thereby revealing similar age effects to those observed in the extension trial in the present study, and possibly increasing the likelihood of finding an effect of label condition at 21-months.

### FUTURE DIRECTIONS

The results of the extension trial suggest that at 21-months, individual infants’ transfer can be facilitated through the provision of supporting information. Future research could examine whether the same type of supporting information, presented differently, could enhance younger infants’ transfer. For example, it is possible that in the present study, the novelty of the label, the object, and the label-object pairing may have negatively impacted 18-month-olds’ ability to use the label to guide their transfer of information. A training study could examine whether increasing the familiarity of the target object and label, and strengthening the association between them by providing multiple exposures to the object-label pairing over the course of a week, would result in facilitated transfer of the object’s non-obvious property at test. It is also possible that labels simply do not enhance transfer from 2D representations to 3D objects prior to 21-months of age. If this were the case, it would be important to investigate whether other kinds of information might facilitate slightly younger infants’ learning and transfer. For example, additional research could examine the effects of highlighting the symbolic relationship between pictures and objects (e.g., Callaghan and Rankin, 2002) or the effects of presenting infants with multiple different-colored exemplars of the target object while teaching them about the objects’ non-obvious property (e.g., Gentner and Namy, 1999, 2004). This additional research could help to clarify for parents and educators the ideal manner in which to present pictorial information to younger infants.

### CONCLUSION

In summary, the present study provides insight into the development of the ability to transfer information from picture books to the real world. The results of the present study extend previous research by demonstrating that shared labels can facilitate the transfer of complex information in infants just before their second birthday. Importantly, this facilitation was not observed in a group of infants only three months younger. Developmental changes in the ability to apply naming information to the task of transferring complex information suggests that parents of infants 21-months and older might be able to scaffold infants’ transfer from picture books by providing shared labels for depicted and real-world objects, but that the same educational strategy may not result in comparable facilitative effects for younger infants.

## AUTHOR CONTRIBUTIONS

Melanie Khu conducted this research in partial fulfillment of the requirements for the M.Sc. degree, under the supervision of Susan A. Graham. Data from this experiment were included in Melanie Khu’s M.Sc. thesis, submitted to the University of Calgary. Patricia A. Ganea was involved in the conception of the project, as well as manuscript preparation.

## Conflict of Interest Statement

The authors declare that the research was conducted in the absence of any commercial or financial relationships that could be construed as a potential conflict of interest.

## Footnotes

1 A condition in which 18-month-olds were presented with the label and object property information simultaneously similarly failed to find a facilitative effect of naming (Keates, 2010). An additional condition, in which 18-month-olds were reminded of the object’s label prior to testing, also found no facilitation of transfer (Khu et al., 2012). Accordingly, the manner in which naming information is presented does not appear to be responsible for the lack of facilitation observed at 18-months in the present study.
